# Natural variation and selection in *GmSWEET39* affect soybean seed oil content

**DOI:** 10.1111/nph.16250

**Published:** 2019-11-14

**Authors:** Long Miao, Songnan Yang, Kai Zhang, Jianbo He, Chunhua Wu, Yanhua Ren, Junyi Gai, Yan Li

**Affiliations:** ^1^ State Key Laboratory of Crop Genetics and Germplasm Enhancement, National Center for Soybean Improvement Key Laboratory for Biology and Genetic Improvement of Soybean (General, Ministry of Agriculture) Jiangsu Collaborative Innovation Center for Modern Crop Production Nanjing Agricultural University Nanjing 210095 China

**Keywords:** genetic improvement, *GmSWEET39*, natural variation, seed oil content, soybean

## Abstract

- Soybean (*Glycine max*) is a major contributor to the world oilseed production. Its seed oil content has been increased through soybean domestication and improvement. However, the genes underlying the selection are largely unknown.
- The present contribution analyzed the expression patterns of genes in the seed oil quantitative trait loci with strong selective sweep signals, then used association, functional study and population genetics to reveal a sucrose efflux transporter gene, *GmSWEET39*, controlling soybean seed oil content and under selection.
- *GmSWEET39* is highly expressed in soybean seeds and encodes a plasma membrane‐localized protein. Its expression level is positively correlated with soybean seed oil content. The variation in its promoter and coding sequence leads to different natural alleles of this gene. The *GmSWEET39* allelic effects on total oil content were confirmed in the seeds of soybean recombinant inbred lines, transgenic Arabidopsis, and transgenic soybean hairy roots. The frequencies of its superior alleles increased from wild soybean to cultivated soybean, and are much higher in released soybean cultivars.
- The findings herein suggest that the sequence variation in *GmSWEET39* affects its relative expression and oil content in soybean seeds, and *GmSWEET39* has been selected to increase seed oil content during soybean domestication and improvement.

## Introduction

Soybean (*Glycine max* L. Merrill) is widely grown in many countries, constituting an important source of edible oil and protein for human and domestic animals (Leamy *et al.*, 2017). Soybean is recognized as the largest oilseed crop, contributing *c.* 61% of the world oilseed production and *c.* 28% of the total global vegetable oil consumption in 2018 (http://www.soystats.com). Soybean oil consists of five prominent fatty acids that comprise 98.4% of the total oil composition (Li *et al.*, 2013): palmitic acid (16:0, PA), stearic acid (18:0, ST), oleic acid (18:1, OL), linoleic acid (18:2, LI) and linolenic acid (18:3, LN). The major unsaturated fatty acids in soybean oil, OL, LI and LN, might play an important role in the prevention and treatment of a number of diseases, including certain cancers (Rose, 1997) and heart disease (Demaison & Moreau, 2002). Therefore, increasing seed oil content has been an important target during soybean breeding.

Cultivated soybean (*G. max*) was domesticated from wild soybean (*G. soja*), despite the loss of genetic diversity and rare alleles, to gain genetic improvement for important agricultural traits (Hyten *et al.*, 2006). Some genetic loci that govern oil content in soybean seeds were likely selected during soybean domestication (from wild soybean to cultivated soybean) and improvement (from landraces to released cultivars), to meet human needs and breeding goals. For example, 53 domestication‐associated and 43 improvement‐associated selective sweeps overlap with the known oil quantitative trait loci (QTL) regions (Zhou *et al.*, 2015), and some major‐effect QTLs are likely subjected to selection (J. Zhang *et al.*, 2018). Identification of the functional genes underlying these loci could help us to understand the molecular basis controlling oil content in soybean seeds during selection, and also shed light on how to further improve soybean seed oil content in future breeding programmes. *GmZF351*, encoding a zinc finger protein, was found under selection during soybean domestication after comparing the sequences of 51 wild soybean and 48 cultivated soybean varieties, and overexpression of *GmZF351* increases the oil content in transgenic soybean seeds (Q. T. Li *et al.*, 2017). More domestication/improvement‐selective genes related to soybean seed oil accumulation need to be discovered.

Sucrose is the primary source of acetyl‐CoA, which serves as the precursor for lipid biosynthesis (Rawsthorne, 2002). Sucrose is produced in photosynthetically active leaves (sources) and then transported to support nonphotosynthetic tissues (sinks) (Sauer, 2007). Sucrose is a relatively large polar compound and relies on sucrose carrier and sucrose efflux transporters (Sugars Will Eventually be Exported Transporter, SWEET) to achieve efficient transport and allocation (Ayre, 2011; Baker *et al.*, 2012). SWEETs are considered important for the maintenance of animal blood glucose concentrations, sugar translocation between plant organs, plant nectar production, plant seed filling, seed size and plant growth (Chen *et al.*, 2010; Chen *et al.*, 2012; Ma *et al.*, 2017; Bezrutczyk *et al.*, 2018). In Arabidopsis, the *sweet11;12;15* triple mutant shows a ‘wrinkled’ seed phenotype including retarded embryo development, reduced seed weight, reduced starch and lipid content (Chen *et al.*, 2015). In soybean genome, there are a total of 52 putative *GmSWEET* genes that expressed in various tissues (Patil *et al.*, 2015). However, the functions of *GmSWEET* genes in soybean have not been characterized in detail.

The present study identified a sucrose efflux transporter gene *GmSWEET39*, which is highly expressed in soybean seeds and associated with seed oil content in 382 diverse cultivated soybean accessions. Its relative expression level is significantly correlated with oil content in soybean seeds. The variations in the promoter (Pro) and coding sequence (CDS) define six haplotypes of *GmSWEET39* in 80 representative cultivated soybean accessions, and the superior alleles are associated with higher seed oil content. The allelic effects of *GmSWEET39* on total oil content were confirmed by soybean recombinant inbred lines (RILs) as well as transgenic Arabidopsis and soybean hairy roots. The frequencies of *GmSWEET39* superior alleles (improving seed oil content) increased from wild soybean to landraces, and reached > 95% in released cultivars. These findings suggest that *GmSWEET39* has been selected to increase the seed oil content during soybean domestication and improvement. The identified superior alleles of *GmSWEET39* and their germplasm carriers would be valuable resources for the genetic improvement of seed oil content in soybean breeding programmes.

## Materials and Methods

### Plant materials and growth conditions

All soybean accessions were obtained from the National Center for Soybean Improvement, Nanjing, China. The core population consisted of 187 landraces and 195 released cultivars, which originated from six soybean eco‐regions including 24 provinces or districts (Supporting Information, Dataset S1), and was grown at the Jiangpu Experimental Station of Nanjing Agricultural University in Jiangsu province, China, in 2012, 2014 and 2015 (two replications within each year). The recombinant inbred line (RIL) population, consisting of 155 lines derived from a cross between NN92‐128 and ZYD2612, with its two parents were grown at the same location in 2014 and 2015 (F_2:11_ and F_2:12_). Above core population and RIL population were grown in row plots (2‐m long, by 0.5‐m row space, 20 plants per row for each accession), and 41 wild soybean accessions were grown in hill plots (1‐m long by 1‐m row space, three plants for each accession) at the same location, each population with a randomized complete block design. Mature seeds were harvested from each replication and used for phenotypic analysis. *Arabidopsis thaliana* ecotype Columbia‐0 (Col‐0) wild‐type was used for transformation; all Arabidopsis plants were grown under the standard growth conditions (D. Li *et al.*, 2017).

### Analyses of the total oil content

The total seed oil content (percentage of oil on seed DW basis) of soybean core population and RIL population was determined by Fourier‐transform near infrared spectrometry (Bruker Vector 22/N, Germany) based on a method published previously (Y. Zhang *et al.*, 2018). For the estimation of total fatty acid (TFA) content in wild soybean, transgenic Arabidopsis and soybean hairy roots, dry seeds (20 mg) of wild soybean, dry seeds (10 mg) and dry leaves (20 mg) of Arabidopsis, and fresh soybean hairy roots (100 mg) were used for fatty acid extraction and detected by gas chromatograph (Thermo Scientific Trace GC Ultra, Waltham, MA, USA) using the internal standard (heptadecanoic acid) in each sample, according to the method described previously (Liu *et al.*, 2014).

### Genotyping and linkage disequilibrium estimation

The 382 soybean accessions were genotyped by RAD‐seq (He *et al.*, 2017). Briefly, soybean genomic DNA was extracted from young leaves following CTAB method and then digested by *Taq *I enzyme to obtain 400–600‐bp‐long DNA fragments. The DNA fragments then were ligated with adapters followed by sequencing using Illumina HiSeq 2000 platform. Paired‐end reads (90 bp in length) were mapped onto the soybean reference genome (*G. max* Williams 82) using Short Oligonucleotide Alignment Program 2 (Soap2) (Li *et al.*, 2009). The software Realsfs was used to call single nucleotide polymorphisms (SNPs) (Korneliussen *et al.*, 2014) and fastphase software was applied to impute missing data (Scheet & Stephens, 2006). After filtering out nonpolymorphic SNPs and SNPs with > 30% missing data, 145 558 high‐quality SNPs were obtained.

A total of 71 293 SNPs with minor allele frequency (MAF) ≥ 0.05 were used for linkage disequilibrium (LD) analysis. The degree of LD between pairwise SNPs was calculated through the Haploview 4.2 software with a 500‐kb sliding window along each chromosome, whereas the LD decay distance was defined as the physical length when *r* ^2^ (the square of the correlation coefficient between pairwise SNPs) dropped to half its maximum value (Barrett *et al.*, 2005). LD blocks were constructed using the *D’* between each pair of SNPs surrounding *GmSWEET39* in Haploview 4.2 software (Barrett *et al.*, 2005).

### Selective sweep analyses

The 71 293 SNPs were used for selective sweep analysis. The fixation index (*F_ST_*) between soybean landraces and released cultivars, and the nucleotide diversity (π) were analyzed using the vcftools package (Danecek *et al.*, 2011). The reduction of diversity (ROD) was calculated as 1 − (π_released cultivars_/π_landraces_) (Cao *et al.*, 2014). The thresholds of *F_ST_* ≥ 0.19 and ROD ≥ 0.84 which corresponded to the top 2% of sites were employed to identify selective sweeps.

### Tissue expression patterns of candidate genes

RNA‐seq data from 14 soybean tissues were downloaded from Soybase (http://soybase.org) to screen candidate genes with higher expression levels in seeds. The raw data were transformed to the fragments per kilobase of transcript per million mapped reads (FPKM) values which were then displayed as heatmaps using R/pheatmap software. To further compare the relative expression levels of candidate genes in soybean accessions with different seed oil content, seeds were harvested at 10, 20, 30 and 40 d after flowering (DAF) from Jindou20 (24.08% oil content) and Maliaodou (17.53% oil content) for RNA‐isolation and quantitative real time (qRT‐)PCR analysis.

### Regional association study (RAS)

A RAS (Sosso *et al.*, 2015) was performed using the average values of seed oil content of 382 accessions across 3 yr and SNPs within the 10‐Mb region surrounding the candidate gene *GmSWEET39*, with the generalized linear model (GLM) and mixed linear model (MLM) in Tassel 5.0 software (Bradbury *et al.*, 2007). The kinship matrix was calculated by Tassel 5.0 and used as the random effect in MLM. The false discovery rate (FDR) of 0.1 was used to define the corresponding *P*‐value threshold for significant association.

### Relative expression levels of *GmSWEET39*

The tissue expression patterns of *GmSWEET39* (*Glyma.15g049200*, GenBank accession no. NM_001357978.1) were confirmed using the roots, stems, leaves of 2‐wk‐old seedlings, pods (2 cm long) and seeds at four developmental stages (10, 20, 30 and 40 DAF) from a soybean accession Kexin4 (22.84% oil content). The relative expression level of *GmSWEET39* also was determined in the seeds (30 DAF) of 80 soybean accessions, which were selected randomly from the RAS panel including those with high, moderate and low seed oil content. The pods at 15 DAF from transgenic Arabidopsis and control lines also were collected to detect the *GmSWEET39* gene expression level.

### RNA isolation and qRT‐PCR

Total RNA was isolated from 100 mg of each tissue sample following the instruction of plant RNA Extract Kit (TIANGEN Biotech, Beijing, China). The cDNA was synthesized with the Prime Script RT Master Mix kit (TaKaRa, Shiga, Japan). All cDNA samples were diluted to an equal concentration using the NanoDrop‐2000 spectrophotometer (Thermo Scientific, Waltham, MA, USA) and used as templates for quantitation in a 20‐μl reaction system with three biological replicates. The *GmUKN1* (*Glyma.12g020500*, GenBank accession no. NM_001254696.2) and *AtACTIN7* (*AT5G09810*, GenBank accession no. NM_121018.4) were chosen as reference genes to normalize the relative expression level of *GmSWEET39* in soybean and Arabidopsis, respectively.

The qRT‐PCR was performed using LightCycler480 System (Roche Diagnostics Ltd, Rotkreuz, Switzerland) with SYBR Premix Ex Taq Kit (TaKaRa). The relative expression of *GmSWEET39* in Arabidopsis was calculated by 2^‐∆Ct^ and in soybean by 2^‐∆∆Ct^ methods (Livak & Schmittgen, 2001). The sequences of all primers are listed in Table S1.

### Subcellular localization

The coding sequence (CDS) of *GmSWEET39* was amplified from Williams 82 according to the reference sequence in NCBI database using gene‐specific primers (Table S1). Subsequently, *GmSWEET39* was in‐fusion expressed with green fluorescent protein (GFP) under the CaMV 35S promoter (35S:*GmSWEET39‐GFP*) in two backbone vectors, pAN580 and pBinGFP4, respectively, and the empty vectors containing 35S:*GFP* were used as controls. 35S:*AtPIP1‐mCherry* was used as the plasma membrane marker (Duan *et al.*, 2016). These vectors were transferred into Arabidopsis protoplasts or *Agrobacterium tumefaciens* EHA105 and then transformed into *Nicotiana benthamiana* leaves (Yoo *et al.*, 2007; Batistic *et al.*, 2010). At 2–3 d after incubation, the subcellular localization of SWEET39 protein was observed in transformed Arabidopsis protoplasts and the epidermal cells of inoculated tobacco leaves with a laser scanning confocal microscope (Zeiss LSM780).

### Sequence variation and population genetic analyses of *GmSWEET39*

The 2.4‐kb genomic DNA of *SWEET39*, comprising 1.6‐kb promoter region and entire CDS, were amplified using specific primers (Table S1) and sequenced from 121 soybean accessions, including 41 wild soybean accessions, 38 soybean landraces and 42 released cultivars, as well as 155 RILs. These DNA sequences were aligned and compared using ClustalX and Mega 5.0 (Larkin *et al.*, 2007; Tamura *et al.*, 2011). Watterson’s estimator (θ), nucleotide diversity (π) and Tajima’s *D* of *GmSWEET39* were calculated by Tajima's Neutrality Test in Mega 5.0 (Tamura *et al.*, 2011).

### Generation of transgenic Arabidopsis and soybean hairy roots

The CDS of *GmSWEET39* was amplified from Maliaodou and Jindou20 using the primers of *SWEET39*‐35S‐CDS1‐F/R and *SWEET39*‐35S‐CDS2‐F/R (Table S1), respectively. These PCR fragments were cloned into the pCAMBIA3301 vector with CaMV 35S promoter to generate 35S:*GmSWEET39^CDS1^* and 35S:*GmSWEET39^CDS2^* constructs. The 2‐kb promoter (Pro) region of *GmSWEET39* from Jindou20 was amplified by *SWEET39*‐Pro3‐F/R (Table S1). The *GmSWEET39^Pro3^* and CaMV 35S promoter were then ligated with the CDS of *GmSWEET39* from Jindou20 to generate *GmSWEET39^Pro3^*:*GmSWEET39^CDS2^* and 35S:*GmSWEET39^CDS2^* constructs using pBinGFP4 as the backbone vector (*GmSWEET39^CDS2^* was expressed in fusion with GFP). One step cloning kit (VAZYME, Nanjing, China) was used for plasmid construction. Subsequently, the vectors in the pCAMBIA3301 backbone were transferred into *A. tumefaciens* EHA105 to transform Arabidopsis plants by the floral dip method (Clough & Bent, 1998), whereas the constructs with pBinGFP4 as backbone vector were transferred into *A. rhizogenes* strain K599 to infect soybean (Kefeng NO.1) cotyledons, in order to obtain transgenic soybean hairy roots according to the method published previously (Kereszt *et al.*, 2007).

### Sudan red 7B staining and soluble sugar quantification

In order to visualize the lipid concentration, 2‐wk‐old Arabidopsis seedlings were drenched in 0.1% (w/v) Sudan red buffer with phenol as solvent. The samples were stained for 5 h at room temperature in darkness, and then the chlorophyll was expelled by washing with 70% (v/v) ethanol for three times. The samples were quickly photographed by a stereo microscope (Olympus MVX10, Tokyo, Japan).

In order to measure the soluble sugar content in Arabidopsis, siliques at 15 DAF were collected and 10 mg of fine powder per sample was used for soluble sugar extraction based on the published method (Bezrutczyk *et al.*, 2018) and the instruction of the Micro Plant Soluble Sugar Content Assay Kit (Solarbio, Beijing, China). The extraction subsequently was subjected to the microplate reader (Tecan infinite M200, Mannedorf, Switzerland) at the wavelength of 620 nm for soluble sugar analysis.

### Statistical analyses

Sas 9.1 and R 3.5.1 were employed to perform the statistical analysis (Littell *et al.*, 2006). ANOVA was performed using Sas software, and broad‐sense heritability (*h^2^*) was calculated as *h^2^* = σ^2^_g_/(σ^2^_g_ + σ^2^_ge_/*n* + σ^2^/nr) (σ^2^_g_, genotypic variance; σ^2^_ge_, genotype by environment interaction;* n*, number of environments; and r, number of replications (Knapp *et al.*, 1985)). Student’s *t*‐test, Welch’s *t*‐test or Wilcoxon test were used to compare the differences between two groups, whereas Duncan’s multiple range test was used to compare the differences among multiple groups.

### Analyses of sequence variation effects and metabolic pathways

The Phytozome database was used to screen candidate genes with sequence variations leading to potential amino acid changes (Goodstein *et al.*, 2012). The Kyoto Encyclopedia of Genes and Genomes (KEGG) pathways were analyzed using clusterprofiler v.3.8.1 (Yu *et al.*, 2012).

## Results

### Identification of *GmSWEET39* as a candidate gene controlling seed oil content during soybean improvement

**Figure 1:**
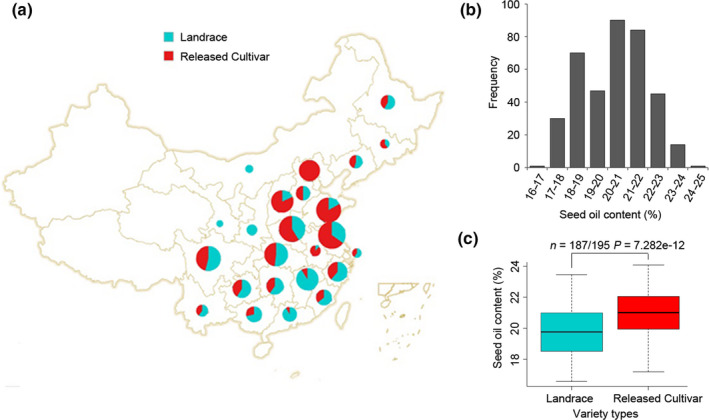
Geographical origins and seed oil contents of 382 cultivated Chinese soybean accessions. (a) Geographical origins of 382 cultivated soybean accessions used in this study. The pie size indicates the number of soybean accessions from each region, sky blue and red colours represent 187 landraces and 195 released cultivars, respectively. (b) Histogram of seed oil content in 382 cultivated soybean accessions. (c) Boxplots of seed oil content in soybean landraces and released cultivars. The central bold line within the box represents the median; box edges indicate the upper and lower quantiles; whiskers show the 1.5 × interquartile range. *P*‐value was determined by two‐tailed two‐sample Wilcoxon test. The average seed oil content across 3 yr is used for (b) and (c).

A core population of 382 diverse cultivated soybean accessions, including 187 landraces and 195 released cultivars originated from a wide range of geographical locations in China (Fig. 1a; Dataset S1), was used to screen the loci associated with soybean improvement from landraces to released cultivars and seed oil content. The broad‐sense heritability of seed oil content is 96% across 3 yr. There are large variations in seed oil content among these 382 diverse soybean accessions, ranging from 15.80% to 24.43% (Fig. 1b). The released cultivars have higher average total seed oil content (Fig. 1c), reflecting the long‐term breeding goal to increase total oil content in soybean seeds.

**Figure 2:**
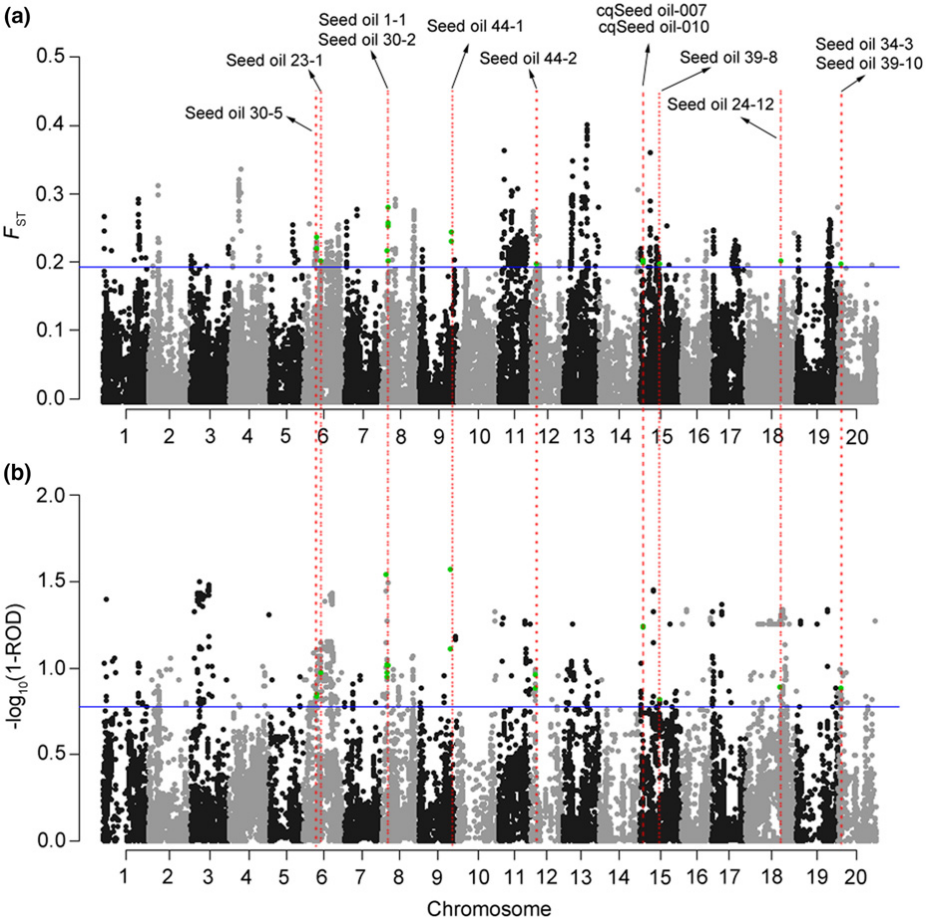
Improvement‐associated selective sweeps and their overlapped seed oil quantitative trait loci (QTL) in 382 cultivated soybean accessions. (a, b) Genetic differentiation (*F*_ST_) and reduction of diversity (ROD) between soybean landraces and released cultivars. The *F*_ST_ and negative log_10_‐transformed (1 – ROD) values are plotted against genome‐wide single nucleotide polymorphism (SNP) positions on 20 chromosomes (only positive values are displayed in b). The thresholds for *F*_ST_ and ROD (corresponding to the top 2% sites in the whole‐genome) are indicated by the horizontal blue lines. Green dots represent the 25 selective sweeps that overlap with the soybean seed oil QTL recorded in Soybase (https://www.soybase.org). The QTL names are shown above the corresponding SNPs and ‘cq’ in front of the QTL names represent confirmed QTL.

**Figure 3:**
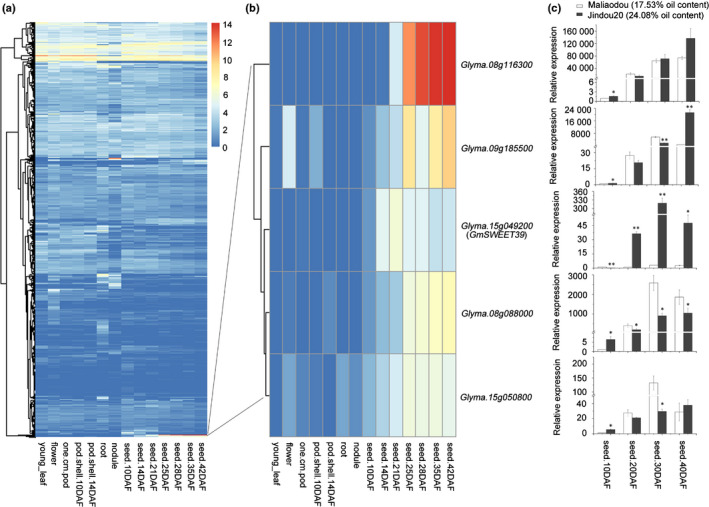
Expression analyses of candidate genes in the genomic regions controlling seed oil content and under selection. (a, b) Heatmaps of candidate gene expression levels in different soybean (*Glycine max*) tissues. The raw reads from RNA‐seq data (www.soybase.org) were transformed to log_2_(FPKM + 1) and only expressed genes are displayed in the heat map. (c) Relative expression of five candidate genes in the seeds of two soybean accessions with low (Maliaodou) and high (Jindou20) seed oil content. The relative expression levels were normalized to that in the seeds at 10 d after flowering (DAF) from Maliaodou. *GmUKN1* was used as the internal control. Error bars indicate SD (*n* = 3). Significant differences between Jindou20 and Maliaodou: *, *P* = 0.05; **, *P* = 0.01 (Student’s *t*‐test). FPKM, fragments per kb of transcript per million mapped reads.

In order to identify the improvement‐associated loci, genome‐wide *F_ST_* and ROD were analyzed in the core population. Following the criteria of *F_ST_* ≥ 0.19 and ROD ≥ 0.84 (corresponding to the top 2% sites in the whole‐genome), 47 SNPs subjected to selective sweeps and 25 SNPs located within the previously mapped QTL regions for seed oil content were identified (Fig. 2a,b; Table S2). The LD decay distance was 786 kb on average across 20 chromosomes with variations on different chromosomes in the population (Fig. S1; Table S3). In order to identify the key candidate genes controlling soybean seed oil content in these loci, the 864 genes within the LD decay distance of these 25 SNPs were examined for their expression levels in different soybean tissues, using the RNA‐seq data from Soybase (https://www.soybase.org/soyseq/). Five genes, *Glyma.08g116300*, *Glyma.09g185500*, *Glyma.15g049200*, *Glyma.08g088000* and *Glyma.15g050800*, are classified into the same cluster with high expression levels in soybean seeds (Fig. 3a,b). Then qRT‐PCR analyses were employed to compare the transcript abundance of these five genes in soybean seeds using two soybean accessions differing in oil content (Fig. 3c). It was found that only one gene, *Glyma.15g049200*, showed much higher relative expression in the seeds of the high seed oil (24.08%) soybean variety Jindou20 than the low seed oil (17.53%) variety Maliaodou. *Glyma.15g049200* was designated as *GmSWEET39* in a previous study on the soybean *SWEET* gene family (Patil *et al.*, 2015), and was predicted to encode a bidirectional sugar transporter. However, the functions of *SWEET* genes in soybean remain to be characterized. Considering that seed oil biosynthesis is directly affected by the accumulation of sucrose in sink tissues, therefore, *GmSWEET39* was selected as a candidate gene controlling seed oil content during soybean improvement.

**Figure 4:**
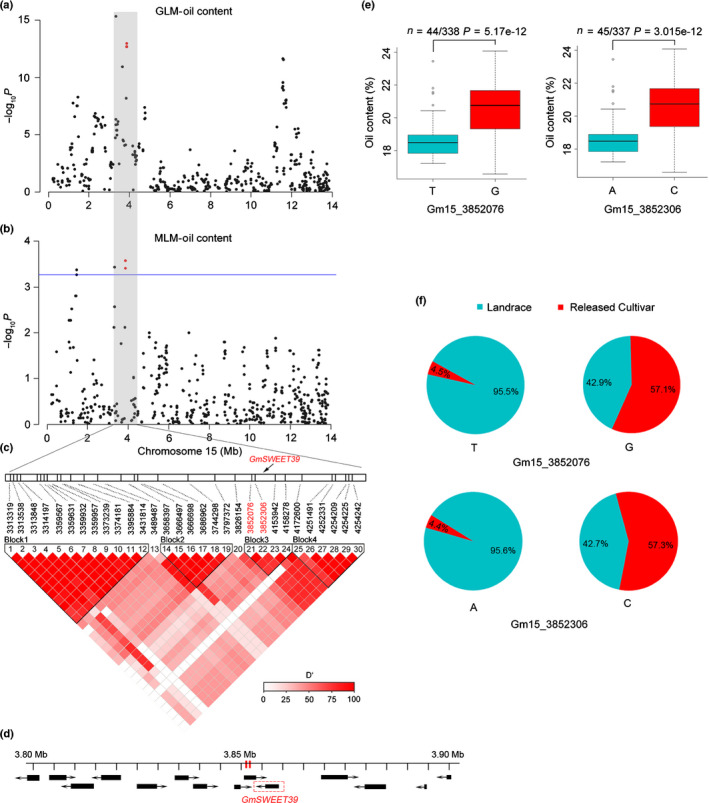
Regional association study (RAS) and allele distribution analysis in 382 cultivated soybean (*Glycine max*) accessions. (a, b) Manhattan plots of RAS on soybean seed oil content by a generalized linear model (GLM, a) or mixed linear model (MLM, b). Negative log_10_‐transformed *P*‐values are plotted against single nucleotide polymorphisms (SNPs) within a 10‐Mb region surrounding the *GmSWEET39* gene, and the significant threshold (MLM, false discovery rate < 0.1) is indicated by the horizontal blue line. The two SNPs, Gm15_3852076 and Gm15_3852306, which showed significant associations with seed oil content as well as strong selective sweep signals, and also located within the confirmed seed oil quantitative trait loci (QTL) and same linkage disequilibrium (LD) block with *GmSWEET39*, are highlighted in red dots. (c) LD analysis of SNPs surrounding *GmSWEET39*. The physical positions of SNPs are indicated above the LD plot, and the physical location of *GmSWEET39* is marked by a black arrow. (d) The 3.80–3.90 Mb region on chromosome 15 of soybean (Williams 82 reference genome), including 14 predicted genes. Black boxes represent the location of genes and arrows represent gene orientations. The physical positions of Gm15_3852076 and Gm15_3852306 are indicated by red vertical lines and the gene in red box is *GmSWEET39*. (e) Comparison of seed oil content in accessions with different alleles of Gm15_3852076 and Gm15_3852306 by boxplots. The central bold line within the box represents the median; box edges indicate the upper and lower quantiles; whiskers show the 1.5 × interquartile range and points indicate outliers. *P*‐values were determined by two‐tailed two‐sample Wilcoxon tests. The seed oil content is the mean of data over 3 yr. (f) Allele frequencies of Gm15_3852076 and Gm15_3852306 in soybean landraces and released cultivars. SWEET, Sugars Will Eventually be Exported Transporter.

Next, a RAS (Sosso *et al.*, 2015) was performed using the seed oil content and SNPs (MAF ≥ 0.05) within the 10‐Mb region surrounding *GmSWEET39* in 382 cultivated soybean accessions. Two statistical models, GLM and MLM, were used for RAS, and the results showed that GLM had higher inflation of *P*‐values, whereas MLM was better at controlling false positives (Fig. 4a,b). Therefore, the results from MLM were used for further analyses. Six SNPs showed significant (FDR < 0.1) association with total seed oil content (Fig. 4b). Two of them, Gm15_3852076 and Gm15_3852306, located within the previously confirmed seed oil QTL regions (cqSeed oil‐007 and cqSeed oil‐010) and showed strong selective sweep signals (Table S2). In addition, these two SNPs are within 4.5‐kb distance from the physical location of *GmSWEET39* and exhibited strong LD with *GmSWEET39* (Fig. 4c,d). Amongst this RAS population, soybean accessions carrying the Gm15_3852076‐G or Gm15_3852306‐C allele have significantly higher average oil content than those with the Gm15_3852076‐T or Gm15_3852306‐A allele (Fig. 4e), and the frequencies of Gm15_3852076‐G and Gm15_3852306‐C alleles are higher in released cultivars than landraces (Fig. 4f). Taken together, these results suggest that *GmSWEET39* is a strong candidate gene in this seed oil content associated region subjected to selection.

### Expression pattern of *GmSWEET39* and subcellular location of its protein

**Figure 5:**
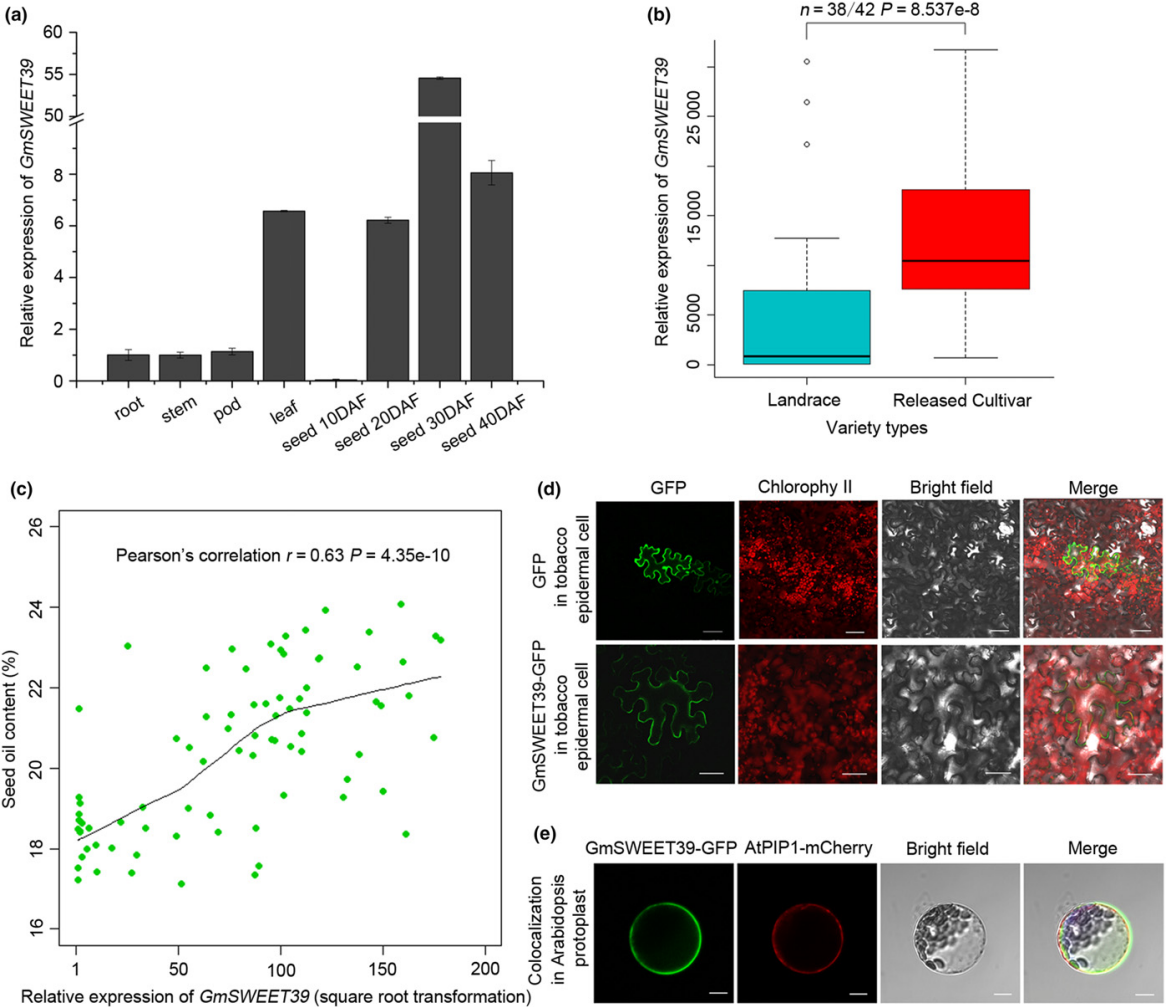
Expression patterns of *GmSWEET39* and the subcellular localization of its protein. (a) *GmSWEET39* expression in various tissues of soybean (*Glycine max*) variety Kexin4. The root, stem and leaf samples were collected from 2‐wk‐old seedlings, whereas the pods in 2‐cm length and seeds at four developmental stages were collected at 10, 20, 30 and 40 d after flowering (DAF). The level of *GmSWEET39* expression was normalized to that in stems and *GmUKN1* was used as the internal control. Error bars indicate SD (*n* = 3). (b) Boxplots of *GmSWEET39* expression levels in the seeds (30 DAF) of landraces (*n* = 38) and released cultivars (*n* = 42). The levels of *GmSWEET39* expression were normalized to that in Wandouzao which contains 17.21% of seed oil content. *GmUKN1* was used as the internal control. The central bold line within the box represents the median; box edges indicate the upper and lower quantiles; whiskers show the 1.5 × interquartile range and points indicate outliers. *P*‐values were determined by two‐tailed two‐sample Wilcoxon test. (c) Correlation analysis between seed oil content and *GmSWEET39* expression level in the seeds (30 DAF) of 80 soybean accessions as shown in (b). The relative expression level of *GmSWEET39* was square root‐transformed. *r* and *P* values were determined by Pearson’s correlation, and the trend line was drawn using the locally weighted scatterplot smoothing (LOWESS) method. (d) Transient expression of green fluorescent protein (GFP) protein or GmSWEET39‐GFP fusion protein under the control of CaMV 35S promoter in tobacco cells. Bars, 50 μm. (e) Subcellular co‐localization of transiently expressed GmSWEET39‐GFP fusion protein with a plasma membrane marker (AtPIP1) in Arabidopsis protoplasts. Bars, 10 μm. SWEET, Sugars Will Eventually be Exported Transporter.

The tissue expression pattern of *GmSWEET39* was verified by qRT‐PCR. As shown in Fig. 5a, *GmSWEET39* was highly expressed in soybean seeds and leaves, with the highest level in seeds at 30 DAF. Next, the expression levels of *GmSWEET39* were investigated in the seeds at 30 DAF among a representative population composed of 80 accessions with high, moderate and low seed oil content, including 38 soybean landraces and 42 released cultivars (Fig. 5b; Table S4). The average expression level of *GmSWEET39* in released cultivars was significantly higher than that in landraces (Fig. 5b), which is consistent with the pattern of seed oil content (Fig. 1c). Further analysis showed that the relative expression level of *GmSWEET39* was positively correlated (*r* = 0.63, *P* = 4.35 × 10^‐10^) with seed oil content (Fig. 5c).

In order to reveal the subcellular localization of GmSWEET39 protein, *GmSWEET39* was expressed in fusion with GFP in tobacco leaves. As shown in Fig. 5d, the GmSWEET39‐GFP fusion protein was localized in plasma membrane. The transient expression of GmSWEET39 in Arabidopsis protoplasts confirmed its plasma membrane localization (Fig. 5e), as indicated by that GmSWEET39‐GFP co‐localized with AtPIP1‐mCherry, a marker for plasma membrane protein (Duan *et al.*, 2016).

### Natural variation and population genetic analyses of *GmSWEET39* gene in soybean landraces and released cultivars

**Figure 6:**
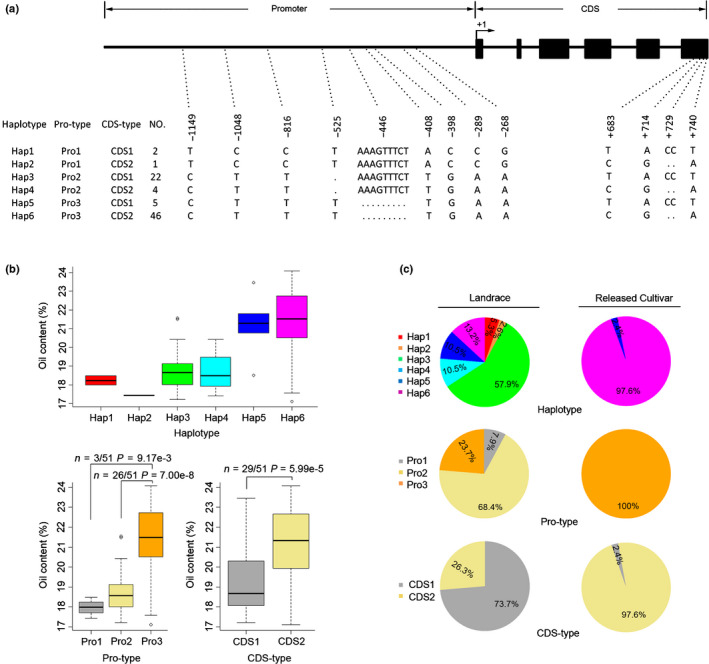
Sequence and allelic variation in *GmSWEET39* among cultivated soybean (*Glycine max*). (a) Sequence and allelic variations in *GmSWEET39* (*c*. 2.4‐kb region) including promoters and coding sequence (CDS) from 38 soybean landraces and 42 released cultivars. Hap1‐Hap6 represents six different haplotypes of *GmSWEET39*; Pro‐type represents different promoter (*c*. 1.6‐kb) types of *GmSWEET39*, CDS‐type represents different CDS of *GmSWEET39*, and NO. represents the number of soybean accessions for each type. The position of start codon is considered as + 1. In CDS region, the black boxes represent exons whereas the lines between boxes represent introns. Nucleotide polymorphisms are displayed at their corresponding positions. (b) Comparison of seed oil content between soybean accessions with different haplotypes, Pro‐types and CDS‐types of *GmSWEET39*, respectively. The seed oil content is the mean of data over 3 yr. The central bold line within the box represents the median; box edges indicate the upper and lower quantiles; whiskers show the 1.5 × interquartile range and points indicate outliers. *P*‐values were determined by two‐tailed two‐sample Wilcoxon tests. (c) Allelic frequencies of *GmSWEET39* in 38 soybean landraces and 42 released cultivars. SWEET, Sugars Will Eventually be Exported Transporter.

The sequence variation of *GmSWEET39* was investigated in the representative population of 38 soybean landraces and 42 released cultivars. *GmSWEET39* contains six exons and five introns, encoding a 249‐amino acid protein, which is predicted to have a molecular mass of 27.64 kDa. A *c.* 2.4‐kb region of *GmSWEET39* was sequenced including its Pro and CDS. A total of 10 SNPs and three InDels (insertions and deletions) were found in this 2.4‐kb region, including seven SNPs + 2 InDels in the Pro and three SNPs + 1 InDel in the CDS (Fig. 6a). Based on these 13 polymorphic sites within *GmSWEET39*, a total of six haplotypes (Hap1–Hap6), from the combination of three Pro types and two CDS types, were identified (Fig. 6a). The effects of *GmSWEET39* haplotypes, Pro and CDS types on soybean seed oil content were then analyzed. The average seed oil content of Hap5 and Hap6 type accessions was generally higher than those in Hap1 to Hap4 types, and that of Hap6 wass slightly higher than Hap5 types. For Pro and CDS types, the average seed oil content of Pro3 or CDS2 type accessions were significantly higher than those in Pro1, Pro2 or CDS1 types, respectively (Fig. 6b). These results suggest that Hap6, Pro3 and CDS2 are the potential superior alleles of *GmSWEET39* that might improve soybean seed oil content.

A further comparison of the distribution of these *GmSWEET39* alleles in soybean landraces and released cultivars revealed several other findings (Fig. 6c). First, soybean landraces have greater allelic diversity in *GmSWEET39* than released cultivars. Landraces covered all six haplotypes, three Pro types and two CDS types, but released cultivars have only two haplotypes, one Pro type and two CDS types. Secondly, soybean landraces have a lower proportion of potential superior alleles of *GmSWEET39* than released cultivars. The frequencies of three types of potential superior alleles of *GmSWEET39*, Hap6, Pro3 and CDS2, in landraces are 13.2% (five of 38), 23.7 (nine of 38) and 26.3% (10 of 38), respectively; whereas these frequencies in released cultivars are 97.6% (41 of 42), 100% (42 of 42) and 97.6% (41 of 42), respectively (Fig. 6c). These observations suggest that *GmSWEET39* gene was likely selected during soybean improvement from landraces to released cultivars. To confirm this hypothesis, Watterson’s estimator (θ), nucleotide diversity (π) and Tajima’s *D* were calculated in *GmSWEET39*. The *GmSWEET39* sequences showed lower θ and π values in released cultivars (θ = 0.000313 and π = 0.000064) compared with those in landraces (θ = 0.001068 and π = 0.001005), indicating a decreased genetic diversity in *GmSWEET39* from soybean landraces to released cultivars. Meanwhile, the Tajima’s *D* for *GmSWEET39* gene in soybean landraces and released cultivars is −0.177 and −1.712, respectively. These results indicate that *GmSWEET39* has been selected during soybean improvement and that the potential superior alleles of *GmSWEET39* tend to be fixed as the preferable types in released cultivars.

### Effect of *GmSWEET39* superior alleles on oil content in transgenic Arabidopsis seeds and soybean hairy roots

**Figure 7:**
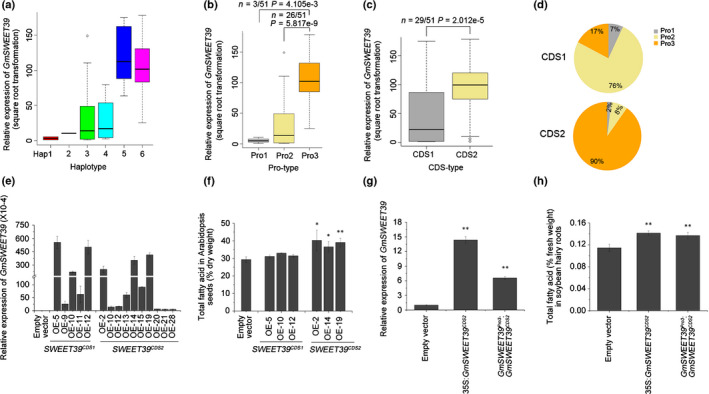
Allelic effects of *GmSWEET39* in the representative cultivated soybean (*Glycine max*) population, transgenic Arabidopsis and soybean hairy roots. (a–c) Comparison of *GmSWEET39* expression levels between soybean accessions containing different haplotypes (Hap), promoters (Pro) and coding sequences (CDS) of *GmSWEET39*, respectively. The dataset is the same as that in Fig. 5(c). The central bold line within the box represents the median; box edges indicate the upper and lower quantiles; whiskers show the 1.5 × interquartile range and points indicate outliers. *P*‐values were determined by two‐tailed two‐sample Wilcoxon tests. (d) Frequencies of different *GmSWEET39* Pro‐types in two CDS groups among 80 cultivated soybean accessions. (e) Expression levels of *GmSWEET39* ^CDS1^ and *GmSWEET39* ^CDS2^ in transgenic Arabidopsis. *AtACTIN7* was used as the internal control and the relative expression of *GmSWEET39* was calculated by 2^‐∆Ct^ (∆Ct = Ct*_GmSWEET39_* − Ct*_AtACT7_*). (f) Total fatty acid (TFA) content in Arabidopsis seeds. Three independent transgenic lines overexpressing (OE) *GmSWEET39* ^CDS1^ or *GmSWEET39* ^CDS2^ were analyzed. (g) Relative expression of *GmSWEET39* in transgenic soybean hairy roots. CaMV 35S promoter or *SWEET39* ^Pro3^ were used to express *GmSWEET39*. *GmUKN1* was used as the internal control and the relative expression of *GmSWEET39* was normalized to that in soybean hairy roots transformed by the empty vector. (h) The TFA content in soybean hairy roots. Data represent mean ± SD (*n* ≥ 3). Significant differences between *GmSWEET39* transgenic lines and empty vector transgenic lines: *, *P* = 0.05; **, *P* = 0.01 (Student’s *t*‐test). SWEET, Sugars Will Eventually be Exported Transporter.

Considering the positive correlation between seed oil content and relative expression level of *GmSWEET39* (Fig. 5c), the relative expression level of *GmSWEET39* between different haplotypes, Pro and CDS types then was compared. As expected, accessions with the three potential superior alleles (Hap6, Pro3 and CDS2) had higher expression levels of *GmSWEET39* than those containing the other alleles except Hap5, which also showed relatively high level of *GmSWEET39* expression (Fig. 7a–c), but is a rare allele (1/42 = 2.4%) in released cultivars (Fig. 6c). Because the gene expression level is affected mainly by promoter region, the frequencies of three Pro types of *GmSWEET39* were further compared in the two CDS groups. As shown in Fig. 7(d), the major Pro type in CDS1 group is Pro2 (76%), whereas most (90%) CDS2 accessions have Pro3 type. The results indicate that the higher *GmSWEET39* expression in CDS2 group is likely due to the higher proportion of Pro3 in this group than CDS1 group, and the improved seed oil content of soybean accessions with potential superior *GmSWEET39* alleles is likely due to the higher expression level of *SWEET39^CDS2^*.

In order to test this hypothesis, the effects of potential superior alleles on oil traits were verified by transgenic Arabidopsis plants and soybean hairy roots. First, *GmSWEET39^CDS1^* and *GmSWEET39^CDS2^* were overexpressed (OE) using the CaMV 35S promoter in Arabidopsis. Five homozygous Arabidopsis lines containing 35S:*GmSWEET39^CDS1^* and 10 homozygous lines containing 35S:*GmSWEET39^CDS2^* were obtained in the T_3_ generation (Figs 7e, S2a). Then three lines with higher *GmSWEET39* expression levels for each CDS type (OE‐5, OE‐10 and OE‐12 for *GmSWEET39^CDS1^*, and OE‐2, OE‐14 and OE‐19 for *GmSWEET39^CDS2^*) were selected for further analyses (Fig. 7f). In comparison to control (empty vector transgenic lines), the *GmSWEET39^CDS2^* overexpressing lines had significantly higher TFA content in mature seeds and showed an increase of 37% in OE‐2, 24% in OE‐14 and 33% in OE‐19, respectively (Fig. 7f). However, no significant difference was observed between *GmSWEET39^CDS1^* overexpressing lines and control lines (Fig. 7f). Two‐wk‐old Arabidopsis seedlings were further stained with Sudan red 7B, which is used for lipid staining in plant materials (Brundrett *et al.*, 1991), revealing more intensified red colour in *SWEET39^CDS2^* overexpressing plants than control (Fig. S2b). The TFA content also was higher in the leaves of *SWEET39^CDS2^* overexpressing Arabidopsis lines than those of the control plants, with an increase of 28% in OE‐2, 11% in OE‐14 and 14% in OE‐19, respectively (Fig. S2c). These results showed that *SWEET39^CDS2^* allele is superior to *SWEET39^CDS1^* for increasing seed oil content. Next, the effects of superior alleles of *GmSWEET39^CDS2^* and *GmSWEET39^pro3^*:*GmSWEET39^CDS2^* (which is Hap6) on oil content in soybean hairy roots were tested using the soybean variety Kefeng No.1, which contains Pro3 and CDS1 type of *GmSWEET39*. Transgenic soybean hairy roots were generated containing 35S:*GmSWEET39^CDS2^* or *GmSWEET39^Pro3^*:*GmSWEET39^CDS2^* (with pBinGFP4 as the backbone vector). The positive transgenic soybean hairy roots were confirmed by GFP signal under fluorescence microscope and used for further analyses. *GmSWEET39* was overexpressed in 35S:*GmSWEET39^CDS2^* and *GmSWEET39^Pro3^*:*GmSWEET39^CDS2^* transgenic lines compared with the control (Fig. 7g). The TFA in soybean hairy roots of 35S:*GmSWEET39^CDS2^* and *GmSWEET39^Pro3^*:*GmSWEET39^CDS2^* was significantly higher than that in the roots containing empty vector (Fig. 7h). These results confirmed that overexpression of the superior allele *GmSWEET39^CDS2^*, or the combination of two superior alleles of *GmSWEET39^Pro3^* and *GmSWEET39^CDS2^* (Hap6), could significantly improve TFA content in Arabidopsis seeds and soybean.

### Confirmation of the *GmSWEET39* allelic effect on seed oil content in soybean RIL population

**Figure 8:**
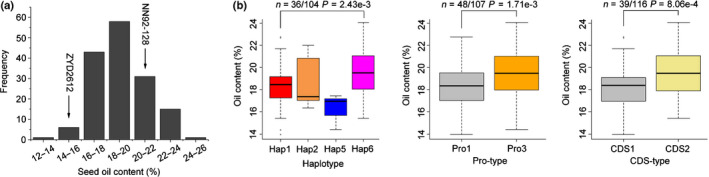
Allelic effect of *SWEET39* on seed oil content in soybean (*Glycine max*) recombinant inbred lines (RILs). (a) Histogram of seed oil content in 155 RILs derived from the cross of NN92‐128 ⅹ ZYD2612. Arrows indicate the seed oil contents of the two parental accessions. (b) Boxplots of seed oil content in soybean accessions with different haplotypes (Hap), promoters (Pro) and coding sequences (CDS) of *GmSWEET39*, respectively. The central bold line within the box represents the median; box edges indicate the upper and lower quantiles; whiskers show the 1.5 × interquartile range and points indicate outliers. *P*‐values were determined by two‐tailed two‐sample Welch’s *t*‐tests. SWEET, Sugars Will Eventually be Exported Transporter.

In order to confirm the allelic effect of *SWEET39* on oil content in soybean seeds, the seed oil content and *SWEET39* sequence in the soybean RIL population (155 lines) derived from a cross between NN92‐128 (cultivated soybean) and ZYD2612 (wild soybean) were analyzed. NN92‐128 contains Hap6 (Pro3 + CDS2) type of *SWEET39* and high seed oil content (21.35%), whereas ZYD2612 has Hap1 (Pro1 + CDS1) type of *SWEE39* and low seed oil content (15.66%). The seed oil content in the RIL population ranged from 13.94 to 24.07% (Fig. 8a). Four haplotypes from the combination of two Pro types and two CDS types, including Hap1 (Pro1 + CDS1), Hap2 (Pro1 + CDS2), Hap5 (Pro3 + CDS1) and Hap6 (Pro3 + CDS2), were identified in 155 RILs. RILs containing the Hap6 (NN92‐128 parent) type of *SWEET39* have significantly higher oil content in seeds than those with the Hap1 (ZYD2612 parent) type of *GmSWEET39* (Fig. 8b). Consistent with the observation in natural populations, RILs carrying the Pro3 or CDS2 type of *SWEET39* had significantly higher seed oil content than those with the Pro1 or CDS1 type of *SWEET39*, respectively (Fig. 8b). These results confirm that the superior *SWEET39* alleles are associated with higher seed oil content in soybean, which could be used to improve oil content in soybean breeding program.

### Frequencies of *SWEET39* superior alleles in wild soybean and cultivated soybean

**Figure 9:**
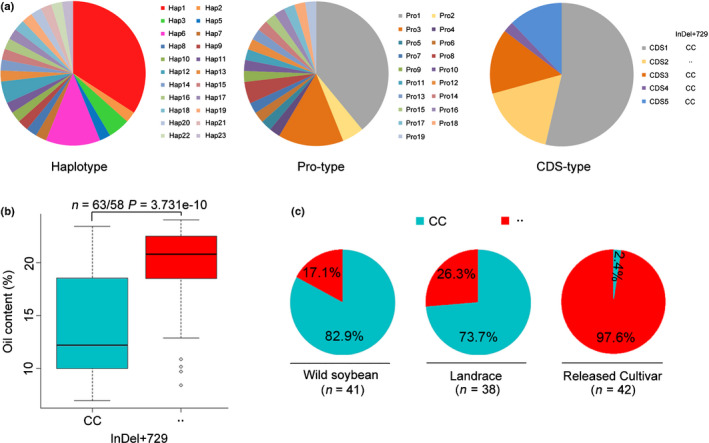
Allelic distribution of *SWEET39* in wild soybean (*Glycine soja*) and cultivated soybean (*G. max*). (a) *GsSWEET39* polymorphisms among 41 wild soybean accessions, including polymorphisms in haplotype (Hap), promoter (Pro) and coding sequence (CDS) of *GmSWEET39*, respectively. (b) Allelic effect of InDel + 729 in *SWEET39* gene on seed oil content in a total of 121 accessions including 41 wild soybean accessions, 38 landraces and 42 released cultivars. The central bold line within the box represents the median; box edges indicate the upper and lower quantiles; whiskers show the 1.5 × interquartile range and points indicate outliers. *P*‐value was determined by two‐tailed two‐sample Wilcoxon test. (c) Allelic distribution of InDel + 729 in wild soybean, landraces and released cultivars. In (b) and (c), sky blue and red colours represent the insertion and deletion of InDel + 729, respectively.

The *SWEET39* gene was further sequenced from 41 wild soybean accessions, revealing more variation in *GsSWEET39* from wild soybean than that in *GmSWEET39* from cultivated soybean, including 22 haplotypes (Hap 4 is absent in these wild soybean accessions), 19 types of Pro and five types of CDS (Fig. 9a; Dataset S2). Due to the high genetic diversity, the frequencies of superior *SWEET39* alleles in wild soybean are only 12% (five of 41) for Hap6, 15% (six of 41) for Pro3 and 17% (seven of 41) for CDS2 types (Fig. 9a), which are lower than those in soybean landraces and much lower than released cultivars (Fig. 6c). Among these polymorphic sites, the InDel + 729 (729 bp downstream of start codon) caused frame shift in the CDS of *SWEET39* (Fig. S3), and, therefore, the allelic effect of InDel + 729 on seed oil content of 121 soybean accessions, including 41 wild soybean accessions, 38 landraces and 42 released cultivars, was further compared. Higher average seed oil content was observed in accessions harbouring the deletion than those harbouring the insertion at InDel + 729 (Fig. 9b). Furthermore, the frequencies of InDel + 729‐deletion increased not only from wild soybean to landraces but also from landraces to released cultivars (Fig. 9c). These results indicated that the deletion at InDel + 729 of *SWEET39* was selected to increase seed oil during soybean domestication and improvement.

## Discussion

Soybean (*G. max*) is the largest oilseed crop in the world (http://www.soystats.com) and breeders have expended a great deal of effort in improving the total oil content in soybean seeds during long‐term selection. However, the genes underlying the natural variation of soybean seed oil content are largely unknown. Here, by analyses of genome‐wide selective sweeps, candidate gene expression and association study on seed oil content in 382 diverse cultivated soybean accessions, it was found that a gene encoding a sugar efflux transporter, *Sugars Will Eventually be Exported Transporter (SWEET)39*, has been selected to increase the seed oil content during soybean breeding history (Figs 2, 3, 4). Recently, *Glyma.15g049200* (*GmSWEET39*) also was considered as a candidate gene for a quantitative trait locus (QTL) controlling seed oil content in a mapping population of chromosome segment substitution lines (Yang *et al.*, 2019). However, its function has not been confirmed. The results herein show that *GmSWEET39* was highly expressed in soybean seeds and its relative expression in the seeds of the high oil content variety Jindou20 was significantly higher than that of low oil content variety Maliaodou (Fig. 3). Furthermore, a positive correlation was found between the relative expression of *GmSWEET39* and seed oil content in 80 cultivated soybean accessions (Fig. 5c). The allelic effects of *GmSWEET39* on oil content were further confirmed by transgenic plants and a soybean recombinant inbred lines (RIL) population (Figs 7e–h, 8). Overexpression of *GmSWEET39^CDS2^* resulted in higher oil content in transgenic plants and soybean hairy roots (Fig. 7e–h); in addition, the combination of *GmSWEET39^Pro3^* and *GmSWEET39^CDS2^* (which is *GmSWEET39^Hap6^*), led to increased oil content in soybean (Figs 7g–h, 8b) (CDS, coding sequence; Hap, haplotype; Pro, promoter). These superior *GmSWEET39* alleles can be used in soybean breeding programmes to improve seed oil content. The two single nucleotide polymorphisms (SNPs), Gm15_3852076 and Gm15_3852306, in the block of linkage disequilibrium (LD) with *GmSWEET39* (Fig. 4c), are correlated with *GmSWEET39* haplotypes (Fig. S4). Soybean accessions with Gm15_3852076‐G or Gm15_3852306‐C have a much higher proportion of potential superior alleles of *GmSWEET39* (Hap6, Pro3 and CDS2) than those with Gm15_3852076‐T or Gm15_3852306‐A in the representative population (Fig. S4). Therefore, Gm15_3852076 and Gm15_3852306 can be used as tagging SNPs for *GmSWEET39* haplotypes in marker‐assisted selection of soybean lines with superior *GmSWEET39* alleles.

The average seed oil content and *GmSWEET39* relative expression in soybean released cultivars are higher than those in landraces (Figs 1c, 5b). In addition, the comparison of Watterson’s estimator (θ), Tajima’s *D* and nucleotide diversity (π) for *GmSWEET39* between soybean landraces and released cultivars implied that *GmSWEET39* has undergone selection. Wild soybean has been considered as the ancestor of cultivated soybean, therefore *GsSWEET39* was further sequenced from 41 wild soybean accessions and it was found that they contained much more variations but lower frequencies of superior alleles in the *SWEET39* gene than cultivated soybean (Fig. 9a). These data suggest that *SWEET39* gene has been selected to increase seed oil content during soybean domestication and improvement. It was also found that wild soybean may contain haplotypes superior to Hap6. For example, accessions with Hap15, Hap19 and Hap20 have higher seed oil content than those containing Hap6 (Dataset S2), but these alleles are rare in the present dataset (only one accession for each haplotype). Therefore, more wild soybean accessions should be further investigated to confirm this possibility.

Members of the SWEET family were found to transport sucrose across the plasma membrane in Arabidopsis and rice (Chen *et al.*, 2012). In *Zea mays*, phloem loading of sucrose was impaired in the *zmsweet13a,b,c* triple mutant (Bezrutczyk *et al.*, 2018). As expected, it was found that the GmSWEET39 protein was localized mainly in the plasma membrane (Fig. 5d,e) and the *GmSWEET39^CDS2^* overexpressing lines had higher soluble sugar content than control in the siliques of Arabidopsis (Fig. S5). In most plants, carbon dioxide is assimilated in mesophyll cells (source) by photosynthesis to produce starch or sucrose, with sucrose as the primary transported form and SWEETs are responsible for sucrose efflux (Doidy *et al.*, 2012; Durand *et al.*, 2018). After being imported into sink organs such as seeds, sucrose can be converted to uridine‐diphosphoglucose (UDPG), fructose or glucose. Glucose can be broken down to pyruvate through glycolysis, and further pyruvate undergoes oxidative decarboxylation to form acetyl‐CoA for fatty acid biosynthesis (Baud & Lepiniec, 2010; Braun *et al.*, 2014). Therefore, overexpression of *GmSWEET39^CDS2^* can increase the soluble sugar content and then lead to enhanced oil synthesis in downstream pathway in Arabidopsis seeds. The detailed mechanism on how GmSWEET39 transports sucrose to seeds should be further investigated using ^14^C‐labeled radio‐tracer, Förster resonance energy transfer (FRET) sensors or other methods (Chen *et al.*, 2010; Chen *et al.*, 2012; Sosso *et al.*, 2015) in future studies.

In rice, the knockout of *OsSWEET11* leads to smaller seeds (Ma *et al.*, 2017). The *sweet4* mutants of both maize and rice also are defective in seed‐filling (Sosso *et al.*, 2015). A recent study proposed that *Glyma.15g049200* (*GmSWEET39*) is likely a candidate gene for the pleiotropic QTL *q100SW15* controlling 100‐seed weight and oil content in soybean (Yang *et al.*, 2019). The 100‐seed weights of the representative 80 accessions from soybean natural population and 155 RILs derived from NN92‐128 × ZYD2612 also were measured. Although in the representative population, the average 100‐seed weight of accessions with superior alleles (Pro3 or CDS2 types of *GmSWEET39*) was higher than that of accessions having Pro1, Pro2 or CDS1 types, there was no significant difference in *GmSWEET39* allelic effect on 100‐seed weight among the RIL population (Fig. S6a–f). In addition, overexpression of *GmSWEET39^CDS1^* or *GmSWEET39^CDS2^* did not cause significant changes in the seed weight of Arabidopsis (Fig. S6g). Therefore, this study showed that *GmSWEET39* affects mainly seed oil content rather than seed weight. It is possible that the gene/QTL effect differs in different populations due to the different genetic background or epistasis, therefore, the role of *GmSWEET39* in soybean seed weight could be further investigated in different genetic background.

In monocots and dicots, SWEET proteins are classified mainly into three clades (I–III) and the SWEETs in clade III are considered to be responsible for seed filling (Eom *et al.*, 2015; Patil *et al.*, 2015). It is noteworthy that this functional feature has been confirmed by the clade III sucrose transporters *OsSWEET11* and *OsSWEET15* in rice (Ma *et al.*, 2017; Yang *et al.*, 2018). *GmSWEET39* in the present study belongs to the clade II SWEETs subfamily (Patil *et al.*, 2015) and, therefore, it could be other clade III SWEETs controlling 100‐seed weight in soybean. Furthermore, soybean is an ancient polyploidy and nearly 75% of its genes have multiple copies (Schmutz *et al.*, 2010). *GmSWEET24* is identified as a paralogous gene of *GmSWEET39* and also highly expressed in developing seed (Patil *et al.*, 2015), indicating that *GmSWEET24* could have a similar role in controlling seed oil content, which needs to be investigated in future studies.

Selective breeding causes remarkable phenotypic changes in soybean seeds such as oil content. In the present study, the possible loci underwent improvement‐associated selection were identified using reduction of diversity (ROD) and fixation index (*F_ST_*), which have been used to screen selective sweeps in soybean (Chung *et al.*, 2014; Han *et al.*, 2016) and peach (Cao *et al.*, 2014). Under the criteria of *F_ST_* ≥ 0.19 and ROD ≥ 0.84 (*c.* 2% top sites of the genome), 47 putative improvement‐selective SNPs were identified (Table S2), 25 of which overlap with the previously reported QTL for seed oil content. Five SNPs on chromosome 15 have been described as improvement‐selective sweeps underlying oil content in 302 soybean accessions (Zhou *et al.*, 2015), whereas the remaining 42 SNPs are newly identified putative improvement‐selective loci in the present study. Different selective signals were detected in different studies, which are likely due to the use of different populations and different sets of molecular markers.

Among the 864 genes in the 25 loci subjected to selective sweeps and located within the previously mapped seed oil QTL regions (Fig. 2; Table S2), in addition to *GmSWEET39*, the genes with sequence variations leading to amino acid changes might also affect soybean seed oil content. Therefore, these 864 genes were scanned for their sequence variation effect from Phytozome database, and 560 genes were found to have variations leading to potential alterations in amino acid sequences. Kyoto Encyclopedia of Genes and Genomes pathway analysis of these 560 genes showed that 16 genes also might affect oil content; these were predicted to be involved in lipid metabolism‐related pathways, including ‘fatty acid elongation’, ‘lipid metabolism’, ‘fatty acid biosynthesis’, ‘lipid biosynthesis proteins’, ‘alpha‐linolenic acid metabolism’, ‘linoleic acid metabolism’, ‘fatty acid degradation’, ‘pyruvate metabolism’, ‘starch and sucrose metabolism’ and ‘glycerolipid metabolism’ (Table S5). Their roles in fatty acid accumulation should be investigated in follow‐up research. This study showed that the integrated information from selective sweeps, QTL and gene expression analysis can help to identify functional genes underlying important agronomic traits such as seed oil content, which would provide invaluable germplasm resources and gene pools to utilize superior alleles in soybean breeding programmes.

## Author contributions

YL and LM designed the study; JG provided soybean accessions and RAD‐seq data; LM performed the experiments, with the assistance of KZ, CW and YR; LM, SY and JH analyzed the data; YL and LM interpreted the results and wrote the manuscript. All authors read and approved the final manuscript.

## Supporting information

Please note: Wiley Blackwell are not responsible for the content or functionality of any Supporting Information supplied by the authors. Any queries (other than missing material) should be directed to the *New Phytologist* Central Office.

**Dataset S1 **Information of the 382 cultivated accessions used in this study.
**Dataset S2** Seed oil content and *GsSWEET39* polymorphisms in 41 wild soybean accessions.

**Fig. S1 **Linkage disequilibrium (LD) decay distance across 20 chromosomes in 382 cultivated Chinese soybean accessions.
**Fig. S2** Confirmation of *GmSWEET39*‐overexpressing Arabidopsis lines and evaluation of fatty acid content in Arabidopsis seedlings.
**Fig. S3** Comparison of the deduced amino acid sequences of SWEET39 protein between CDS1‐type, CDS2‐type and the reference Williams 82.
**Fig. S4** Frequencies of different *GmSWEET39* Hap, Pro and CDS in the allelic groups of Gm15_3852076 and Gm15_3852306 among 80 cultivated soybean accessions.
**Fig. S5** Soluble sugar content in the siliques of transgenic Arabidopsis.
**Fig. S6** Effect of *GmSWEET39* natural alleles on seed weight.
**Table S1** Primers used in this study.
**Table S2** The 47 SNPs with strong selective sweep signals and their overlapped seed oil QTL.
**Table S3** The LD decay distance on 20 chromosomes in cultivated soybean.
**Table S4** The seed oil content and relative expression of *GmSWEET39* in 80 soybean accessions.
**Table S5** The candidate genes with potential alterations in amino acid sequences in predicted lipid metabolism related KEGG pathways.
