# Supplementary material for: Natural variation and selection in GmSWEET39 affect soybean seed oil content
Source: New Phytol. 2019 Nov 14;225(4):1651–66. doi: 10.1111/nph.16250 (PMC7496907; doi:10.1111/nph.16250)
Supplement: Supplementary file 2 — Fig. S1 Linkage disequilibrium (LD) decay distance across 20 chromosomes in 382 cultivated Chinese soybean accessions. Fig. S2 Confirmation of GmSWEET39‐overexpressing Arabidopsis lines and evaluation of fatty acid content in Arabidopsis seedlings. Fig. S3 Comparison of the deduced amino acid sequences of SWEET39 protein between CDS1‐type, CDS2‐type and the reference Williams 82. Fig. S4 Frequencies of different GmSWEET39 Hap, Pro and CDS in the allelic groups of Gm15_3852076 and Gm15_3852306 among 80 cultivated soybean accessions. Fig. S5 Soluble sugar content in the siliques of transgenic Arabidopsis. Fig. S6 Effect of GmSWEET39 natural alleles on seed weight. Table S1 Primers used in this study. Table S2 The 47 SNPs with strong selective sweep signals and their overlapped seed oil QTL. Table S3 The LD decay distance on 20 chromosomes in cultivated soybean. Table S4 The seed oil content and relative expression of GmSWEET39 in 80 soybean accessions. Table S5 The candidate genes with potential alterations in amino acid sequences in predicted lipid metabolism related KEGG pathways. [file NPH-225-1651-s002.pdf]

Article title: Natural variation and selection in *GmSWEET39* affect soybean seed oil content

Authors: Long Miao, Songnan Yang, Kai Zhang, Jianbo He, Chunhua Wu, Yanhua Ren, Junyi Gai and Yan Li*

Article acceptance date: 2 October 2019

Supporting Figures:

Fig. S1 Linkage disequilibrium (LD) decay distance across 20 chromosomes in 382 cultivated Chinese soybean accessions.

Fig. S2 Confirmation of *GmSWEET39*-overexpressing Arabidopsis lines and evaluation of fatty acid content in Arabidopsis seedlings.

Fig. S3 Comparison of the deduced amino acid sequences of SWEET39 protein between CDS1-type, CDS2-type and the reference Williams 82.

Fig. S4 Frequencies of different *GmSWEET39* haplotypes (Hap), promoters (Pro) and coding sequences (CDS) in the allelic groups of Gm15_3852076 and Gm15_3852306 among 80 cultivated soybean accessions.

Fig. S5 Soluble sugar content in the siliques of transgenic Arabidopsis.

Fig. S6 Effect of *GmSWEET39* natural alleles on seed weight.

Supporting Tables:

Table S1 Primers used in this study.

Table S2 The 47 SNPs with strong selective sweep signals and their overlapped seed oil quantitative trait loci (QTL).

Table S3 Linkage disequilibrium (LD) decay distance on 20 chromosomes in cultivated soybean.

Table S4 The seed oil content and relative expression of *GmSWEET39* in 80 soybean accessions.

Table S5 The candidate genes with potential alterations in amino acid sequences in predicted lipid metabolism related KEGG (Kyoto Encyclopedia of Genes and Genomes) pathways.

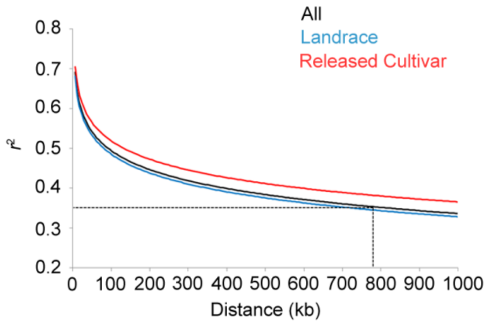

Fig. S1 Linkage disequilibrium (LD) decay distance across 20 chromosomes in 382 cultivated Chinese soybean accessions. The LD decay was estimated by squared correlation coefficient (r^2) of all pairs of SNPs using 500-kb sliding window. The dashed line represents the position where r^2 declined to half of its maximum value in all accessions. All: 382 soybean accessions including landraces and released cultivars; Landrace: 187 accessions; Released Cultivar: 195 accessions.

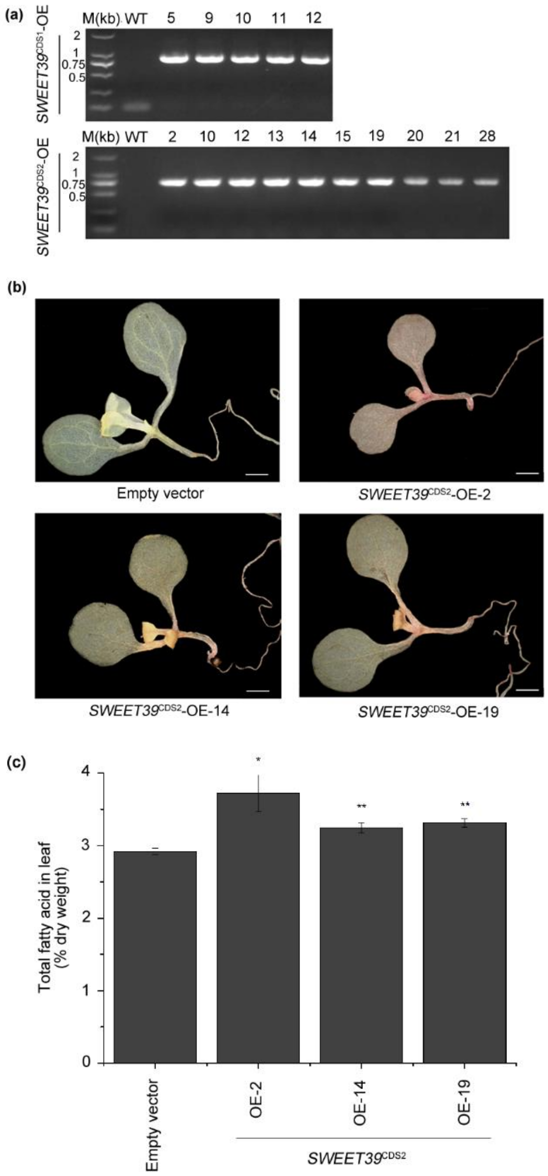

Fig. S2 Confirmation of *GmSWEET39*-overexpressing Arabidopsis lines and evaluation of fatty acid content in Arabidopsis seedlings. Soybean *SWEET39*^{CDS1} and *GmSWEET39*^{CDS2} were overexpressed (OE) by CaMV 35S promoter. (a) Confirmation of transgenic Arabidopsis plants overexpressing two different CDS-types of *GmSWEET39*, using the genomic DNA from Arabidopsis leaves as the template. M, DL2000 DNA marker. WT, Arabidopsis Col-0 wild type plants. The rest lanes represent different homozygous Arabidopsis lines transformed with pCAMBIA3301-35S:*GmSWEET39*^{CDS1} or pCAMBIA3301-35S:*GmSWEET39*^{CDS2}. (b) Fad red 7B staining of two-week-old Arabidopsis seedlings from control lines (empty vector) or *GmSWEET39*^{CDS2} overexpressing lines. Scale bars indicate 890 μm. (c)

Total fatty acid content in Arabidopsis leaves. Data represents mean ± SD (n = 3). * and ** represent significant difference (Student's *t*-test) between *GmSWEET39*^{CDS2} overexpressing lines and empty vector transgenic lines at 0.05 and 0.01 level, respectively.

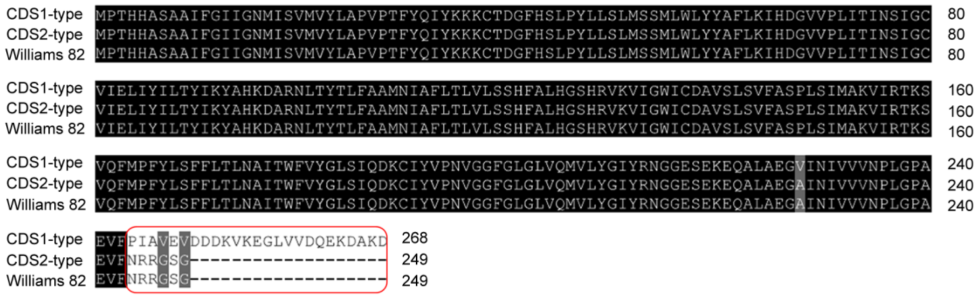

Fig. S3 Comparison of the deduced amino acid sequences of SWEET39 protein between CDS1-type, CDS2-type and the reference Williams 82. Amino acid residue changes are highlighted in red boxes. An insertion of CC in the coding sequence of *GmSWEET39* in CDS1-type resulted in frame shift and the number of amino acid residues increased from 249 to 268 when compared to CDS2-type or Williams 82. CDS: coding sequence.

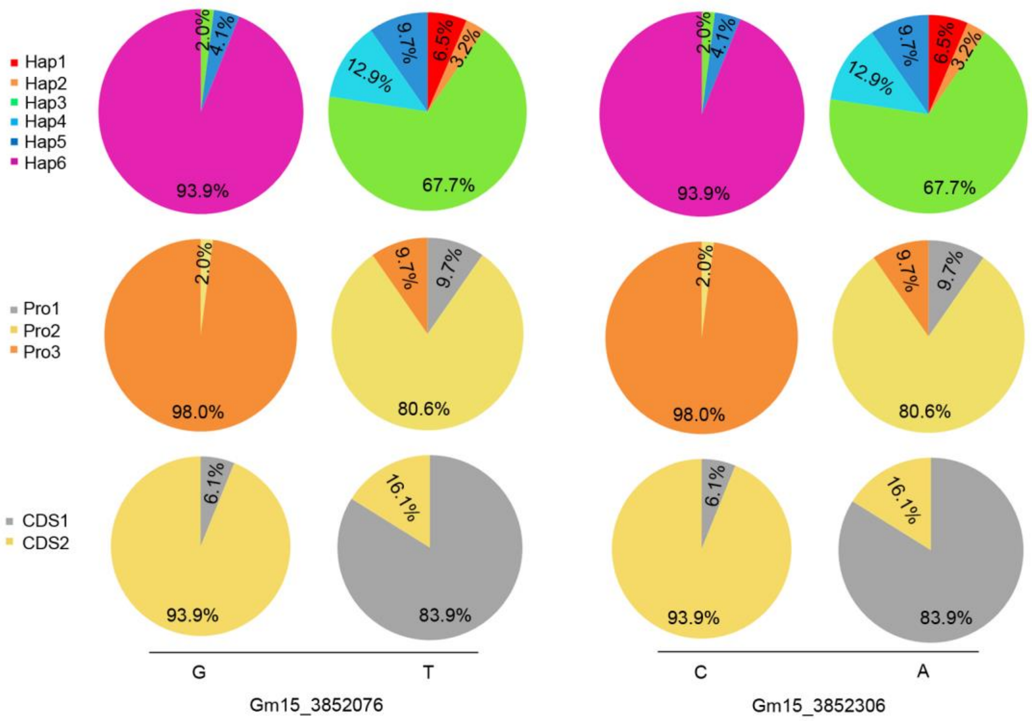

Fig. S4 Frequencies of different *GmSWEET39* haplotypes (Hap), promoters (Pro) and coding sequences (CDS) in the allelic groups of Gm15_3852076 and Gm15_3852306 among 80 cultivated soybean accessions.

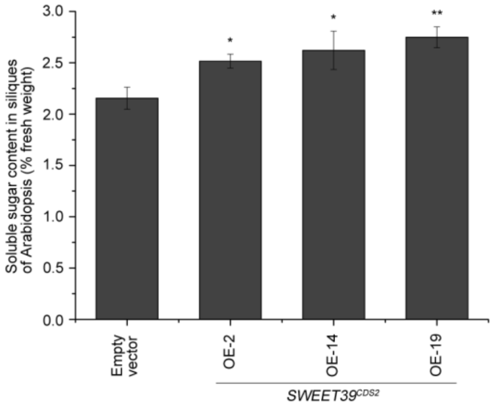

Fig. S5 Soluble sugar content in the siliques of transgenic Arabidopsis. *GmSWEET39*CDS2 was overexpressed (OE) by CaMV 35S promoter and three homozygous lines (OE-2, 14, 19) were compared with the empty vector control. Data represents mean ± SD (n = 3). P values were determined by Student's t-test: *, $P \leqslant 0.05$; **, $P \leqslant 0.01$.

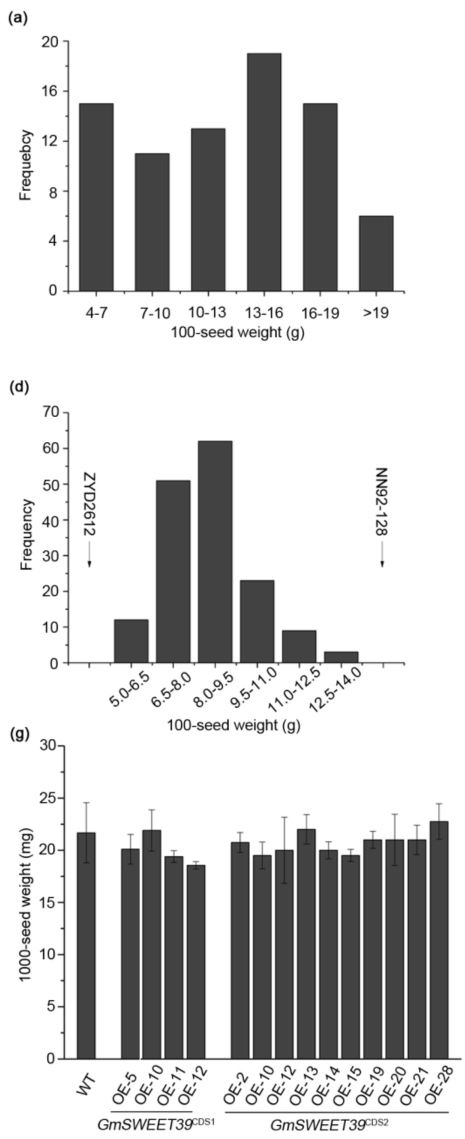

Fig. S6 Effect of *GmSWEET39* natural alleles on seed weight. (a-c) Phenotypic distribution and allelic effect of *GmSWEET39* on 100-seed weight in the representative population of 38 soybean landraces and 42 released cultivars. (d-f) Phenotypic distribution and allelic effect of *GmSWEET39* on 100-seed weight in 155 recombinant inbred lines derived from NN92-128 × ZYD2612. (g) Allelic effect of *GmSWEET39* on 1000-seed weight of Arabidopsis. *GmSWEET39*^{CDS1} and *GmSWEET39*^{CDS2} were overexpressed (OE) by CaMV 35S promoter. Data represents mean ± SD (n = 4). *P* values were determined by two-tailed two-sample Wilcoxon tests (b, c, e, f) or Student's *t*-tests (g).

Table S1. Primers used in this study.

Name	Primer (5' to 3')	Note
GmSWEET39-Seq-CDS-F	GTTGCTCATCACATCCCCCA	cloning *GmSWEET39* CDS for sequencing
GmSWEET39-Seq-CDS-R	GGCGGCCGTAAGCAATTAAA	cloning *GmSWEET39* CDS for sequencing
GmSWEET39-Seq-Pro-F	AAAGGAATACGGCGTGGTCT	cloning *GmSWEET39* Promoter for sequencing
GmSWEET39-Seq-Pro-R	GAAGCAAAGAAACAAGAAGATGAGC	cloning *GmSWEET39* Promoter for sequencing
GmSWEET39-qRT-F	TGGACTGGGGCTAGTTCAGA	qRT-PCR
GmSWEET39-qRT-R	TCCACTTCCTCTGCGATTGA	qRT-PCR
GmUKN1-qRT-F	GCGAATGTTACGGAGGCTCTG	qRT-PCR
GmUKN1-qRT-R	ATCTGAATGGTGGCGGGATCAA	qRT-PCR
AtACTIN7-F	CCTCAGCACCTTCCAACAGATG	qRT-PCR
AtACTIN7-R	AACTCACCACCACGAACCAGAT	qRT-PCR
GmSWEET39-SUB1-F	**AAGTCCGGAGCTAGCTCTAGA**ATGCCCACTCACCATGCTTC	subcellular localization, the bold sequence indicates adaptor for one-step cloning
GmSWEET39-SUB1-R	**GCTCACCATGGATCCCCCGGG**TCCACTTCCTCTGCGATTGAA	subcellular localization, the bold sequence indicates adaptor for one-step cloning
GmSWEET39-SUB2-F	**GAACGATAGGGTACCCCCGGG**ATGCCCACTCACCATGCTTC	subcellular localization and overexpression of CDS2 type of *GmSWEET39* in soybean hairy roots, the bold sequence indicates adaptor for one-step cloning
GmSWEET39-SUB2-R	**GCCCTTGCTCACCATGGATCC**TCCACTTCCTCTGCGATTGAA	subcellular localization and overexpression of CDS2 type of *GmSWEET39* in soybean hairy roots, the bold sequence indicates adaptor for one-step cloning
GmSWEET39-35S-CDS1-F1	**GAACGATAGGGTACCCCCGGG**ATGCCCACTCACCATGCTTC	overexpression of CDS1 type of *GmSWEET39* with 35S promoter in soybean hairy roots, the bold sequence indicates adaptor for one-step cloning
GmSWEET39-35S-CDS1-R1	**GCCCTTGCTCACCATGGATCC**ATCCTTGGCATCCTTCTCTTGA	overexpression of CDS1 type of *GmSWEET39* with 35S promoter in soybean hairy roots, the bold sequence indicates adaptor for one-step cloning

Name	Primer (5' to 3')	Note
GmSWEET39-35S-CDS1-F2	**TCTAGAGGATCTCGAGGCGCGCC**ATGCCCACTCACCATGCTTC	overexpression of CDS1 type of *GmSWEET39* with 35S promoter in Arabidopsis, the bold sequence indicates adaptor for one-step cloning
GmSWEET39-35S-CDS1-R2	**ATTCGAGCTCACTAGTTAATTAA**ATCCTTGGCATCCTTCTCTTGA	overexpression of CDS1 type of *GmSWEET39* with 35S promoter in Arabidopsis, the bold sequence indicates adaptor for one-step cloning
GmSWEET39-35S-CDS2-F1	**GAACGATAGGGTACCCCCGGG**ATGCCCACTCACCATGCTTC	overexpression of CDS2 type of *GmSWEET39* with 35S promoter in soybean hairy roots, the bold sequence indicates adaptor for one-step cloning
GmSWEET39-35S-CDS2-R1	**GCCCTTGCTCACCATGGATCC**TCCACTTCCTCTGCGATTGAA	overexpression of CDS2 type of *GmSWEET39* with 35S promoter in soybean hairy roots, the bold sequence indicates adaptor for one-step cloning
GmSWEET39-35S-CDS2-F2	**TCTAGAGGATCTCGAGGCGCGCC**ATGCCCACTCACCATGCTTC	overexpression of CDS2 type of *GmSWEET39* with 35S promoter in Arabidopsis, the bold sequence indicates adaptor for one-step cloning
GmSWEET39-35S-CDS2-R2	**ATTCGAGCTCACTAGTTAATTAA**TCCACTTCCTCTGCGATTGAA	overexpression of CDS2 type of *GmSWEET39* with 35S promoter in Arabidopsis, the bold sequence indicates adaptor for one-step cloning
GmSWEET39-Pro3-F	**GTTAATTAAGAATTCGAGCTC**TGTTCTTTAATTAAGCGTCAAG	amplification of Pro3 for expression of *GmSWEET39* in soybean hairy roots, the bold sequence indicates adaptor for one-step cloning
GmSWEET39-Pro3-R	**AGGCGCGCC**GATGGATCCTCTGAAGCTATTAGGT	amplification of Pro3 for expression of *GmSWEET39* in soybean hairy roots, the bold sequence indicates adaptor for one-step cloning
GmSWEET39-CDS2-F	**AGGCGCGCC**ATGCCCACTCACCATGCTTCC	amplification of CDS2 for expression of *GmSWEET39* in soybean hairy roots, the bold sequence indicates adaptor for one-step cloning
GmSWEET39-CDS2-R	**GTTGTGTTGAGAATTCTCGAG**TCCACTTCCTCTGCGATTGAA	amplification of CDS2 for expression of *GmSWEET39* in soybean hairy roots, the bold sequence indicates adaptor for one-step cloning

Table S2. The 47 SNPs with strong selective sweep signals and their overlapped seed oil quantitative trait loci (QTL).

SNP[a]	F_{ST}	ROD	$-Log_{10}(1-ROD)$	QTL name[b]	QTL start[c]	QTL end[c]	Marker[d]
Gm06_15836261	0.208	0.85	0.828	–	–	–	–
Gm06_15836458	0.203	0.85	0.823	–	–	–	–
Gm06_15897258	0.230	0.88	0.915	–	–	–	–
Gm06_15936181	0.224	0.88	0.910	–	–	–	–
Gm06_15945565	0.225	0.86	0.843	–	–	–	–
Gm06_15950570	0.224	0.88	0.910	–	–	–	–
Gm06_15950592	0.236	0.88	0.920	–	–	–	–
Gm06_15950608	0.236	0.88	0.920	–	–	–	–
Gm06_15950711	0.236	0.88	0.920	–	–	–	–
Gm06_15950895	0.236	0.88	0.920	–	–	–	–
Gm06_15993488	0.219	0.88	0.905	–	–	–	–
Gm06_15993880	0.219	0.88	0.905	–	–	–	–
Gm06_16025755	0.213	0.90	0.983	–	–	–	–
Gm06_16025975	0.225	0.92	1.106	–	–	–	–
Gm06_16034349	0.219	0.90	0.989	–	–	–	–
Gm06_16208000	0.219	0.85	0.838	Seed oil 30-5	16084033	17173887	BARC-044133-08626/Satt277
Gm06_16255759	0.236	0.86	0.852	Seed oil 30-5	16084033	17173887	BARC-044133-08626/Satt277
Gm06_16255788	0.219	0.85	0.838	Seed oil 30-5	16084033	17173887	BARC-044133-08626/Satt277
Gm06_21335783	0.202	0.89	0.972	Seed oil 23-1	17173887	43950980	Satt277/Satt079
Gm06_21366243	0.202	0.89	0.972	Seed oil 23-1	17173887	43950980	Satt277/Satt079
Gm08_7020722	0.216	0.97	1.540	Seed oil 1-1	5765078	12389879	BARC-039593-07509/BARC-014665-01613
Gm08_8191353	0.202	0.89	0.972	Seed oil 1-1	5765078	12389879	BARC-039593-07509/BARC-014665-01613

SNP[a]	F_{ST}	ROD	$-Log_{10}(1-ROD)$	QTL name[b]	QTL start[c]	QTL end[c]	Marker[d]
				Seed oil 30-2	7885825	9211886	Sat_400/Sat_215
Gm08_8196684	0.280	0.89	0.951	Seed oil 1-1	5765078	12389879	BARC-039593-07509/BARC-014665-01613
				Seed oil 30-2	7885825	9211886	Sat_400/Sat_215
Gm08_8198033	0.280	0.89	0.951	Seed oil 1-1	5765078	12389879	BARC-039593-07509/BARC-014665-01613
				Seed oil 30-2	7885825	9211886	Sat_400/Sat_215
Gm08_8227016	0.280	0.89	0.951	Seed oil 1-1	5765078	12389879	BARC-039593-07509/BARC-014665-01613
				Seed oil 30-2	7885825	9211886	Sat_400/Sat_215
Gm08_8261684	0.258	0.91	1.023	Seed oil 1-1	5765078	12389879	BARC-039593-07509/BARC-014665-01613
				Seed oil 30-2	7885825	9211886	Sat_400/Sat_215
Gm08_8316816	0.252	0.90	1.019	Seed oil 1-1	5765078	12389879	BARC-039593-07509/BARC-014665-01613
				Seed oil 30-2	7885825	9211886	Sat_400/Sat_215
Gm08_8316821	0.252	0.90	1.019	Seed oil 1-1	5765078	12389879	BARC-039593-07509/BARC-014665-01613
				Seed oil 30-2	7885825	9211886	Sat_400/Sat_215
Gm08_8444816	0.252	0.90	1.019	Seed oil 1-1	5765078	12389879	BARC-039593-07509/BARC-014665-01613
				Seed oil 30-2	7885825	9211886	Sat_400/Sat_215
Gm09_38608275	0.231	0.92	1.112	Seed oil 44-1	34198309	39067035	Sat_044/BARC-044609-08738
Gm09_38608318	0.244	0.97	1.573	Seed oil 44-1	34198309	39067035	Sat_044/BARC-044609-08738
Gm12_6094884	0.197	0.89	0.965	Seed oil 44-2	1682557	33808502	Satt353/Satt629
Gm12_6117997	0.197	0.87	0.883	Seed oil 44-2	1682557	33808502	Satt353/Satt629
Gm12_6127001	0.197	0.87	0.883	Seed oil 44-2	1682557	33808502	Satt353/Satt629
Gm15_1387805	0.214	0.85	0.833	—	—	—	—
Gm15_1416172	0.214	0.85	0.833	—	—	—	—
Gm15_1416203	0.214	0.85	0.833	—	—	—	—
**Gm15_3852076**	0.198	0.94	1.234	cqSeed oil-007	3298019	3966810	BARC-039687-07541/BARC-042349-08247

SNP[a]	F_{ST}	ROD	$-Log_{10}(1-ROD)$	QTL name[b]	QTL start[c]	QTL end[c]	Marker[d]
				cqSeed oil-010	3298019	3966810	BARC-039687-07541/BARC-042349-08247
**Gm15_3852306**	0.204	0.94	1.242	cqSeed oil-007	3298019	3966810	BARC-039687-07541/BARC-042349-08247
				cqSeed oil-010	3298019	3966810	BARC-039687-07541/BARC-042349-08247
Gm15_24109364	0.197	0.85	0.818	Seed oil 39-8	9396584	39682051	Sat_273/Satt452
Gm17_1935996	0.230	0.88	0.915	–	–	–	–
Gm17_1985584	0.236	0.88	0.920	–	–	–	–
Gm17_2000636	0.247	0.88	0.929	–	–	–	–
Gm17_2020292	0.224	0.88	0.910	–	–	–	–
Gm18_42297494	0.202	0.87	0.889	Seed oil 24-12	30921715	50379761	Sat_088/Satt400
Gm18_42297515	0.202	0.87	0.889	Seed oil 24-12	30921715	50379761	Sat_088/Satt400
Gm20_3628883	0.197	0.87	0.883	Seed oil 39-10	1888712	3915962	Satt562/Satt614
				Seed oil 34-3	2053507	4661633	BARC-021343-04041/BARC-024577-05301

[a] Gm15_3852076 and Gm15_3852306 in bold were detected by both selective sweep analyses and regional association study.

[b] Reported seed oil QTL in Soybase (https://www.soybase.org/). 'cq' in front of the QTL names represent confirmed QTL.

[c] The physical location of each QTL.

[d] Markers associated with the reported QTL.

The symbol "-" indicates no recorded QTLs in Soybase.

Table S3. Linkage disequilibrium (LD) decay distance on 20 chromosomes in cultivated soybean.

Chromosome	LD decay distance[a]
1	784 kb
2	320 kb
3	343 kb
4	250 kb
5	161 kb
6	201 kb
7	155 kb
8	720 kb
9	617 kb
10	734 kb
11	321 kb
12	147 kb
13	105 kb
14	727 kb
15	300 kb
16	332 kb
17	616 kb
18	452 kb
19	1300 kb
20	178 kb

[a] LD decay distance was determined when r^2 dropped to half of its maximum value.

Table S4. The seed oil content and relative expression of *GmSWEET39* in 80 soybean accessions.

Individual code	NJAU code	Accession type	Oil content (%)	Relative expression of *SWEET39* in seeds at 30 DAF
L001	N24296	Landrace	18.38	3.69
L007	N05283.2	Landrace	21.49	3.24
L012	N04421	Landrace	21.28	4039.61
L019	N05433.2	Landrace	18.44	4.06
L023	N23576	Landrace	18.50	1157.40
L028	N03073	Landrace	19.12	3.53
L037	N21056	Landrace	17.53	1.52
L050	N24150	Landrace	18.01	317.37
L059	N24309	Landrace	19.28	2.46
L060	N20941	Landrace	17.31	7661.09
L061	N24274	Landrace	21.55	22226.61
L069	N24139	Landrace	18.86	2.10
L071	N02483	Landrace	17.80	9.65
L079	N5076.1	Landrace	20.31	7451.59
L081	N05050	Landrace	17.43	107.39
L082	N24049	Landrace	18.08	99.04
L083	N4411	Landrace	19.03	1055.22
L095	N08612	Landrace	18.72	2.67
L103	N21196	Landrace	20.99	5505.53
L106	N04753.1	Landrace	18.30	2396.42
L108	N22395	Landrace	20.70	9195.21
L123	N04815	Landrace	20.43	6338.83
L126	N24608	Landrace	17.21	1.00
L138	N04869.2	Landrace	20.42	12189.35
L144	N22798.1	Landrace	21.57	7608.17
L145	N05321.22	Landrace	21.39	12765.83
L149	N24611	Landrace	18.64	9.34
L150	N10384	Landrace	19.01	3005.38
L153	N24284	Landrace	18.51	39.03
L154	N3172	Landrace	21.80	26432.04
L159	N24617	Landrace	17.99	28.25
L161	N02786	Landrace	20.77	30573.63
L164	N9962	Landrace	17.85	887.33
L167	N09465	Landrace	18.66	477.71
L169	N3188	Landrace	17.40	758.32
L176	N04566	Landrace	17.12	2652.86

Individual code	NJAU code	Accession type	Oil content (%)	Relative expression of *SWEET39* in seeds at 30 DAF
L179	N24286	Landrace	23.45	12619.20
L180	N24619	Landrace	18.49	1.00
R005	N25107	Released Cultivar	19.33	10369.08
R007	N25455	Released Cultivar	20.73	2435.50
R008	N25449	Released Cultivar	22.48	6904.56
R011	N25343	Released Cultivar	23.40	20499.96
R016	N25349	Released Cultivar	21.99	12677.65
R019	N25301	Released Cultivar	24.08	25238.41
R025	N25269	Released Cultivar	18.51	7786.00
R026	N25278	Released Cultivar	20.71	9453.72
R028	N20827.000	Released Cultivar	21.65	21519.15
R032	T614836-37	Released Cultivar	22.49	4048.95
R034	N09144.000	Released Cultivar	22.74	14131.89
R045	N01197.210	Released Cultivar	18.42	4815.04
R052	N25298	Released Cultivar	21.32	9585.69
R056	N21295.000	Released Cultivar	19.41	22484.87
R064	N23748.000	Released Cultivar	18.36	26068.14
R069	N23915.000	Released Cultivar	20.16	3884.02
R071	N25134	Released Cultivar	22.52	18907.44
R084	N25350	Released Cultivar	23.30	30928.87
R087	N25468	Released Cultivar	21.60	8659.09
R091	N25448	Released Cultivar	23.29	10513.82
R092	N25469	Released Cultivar	20.80	7643.41
R093	N25503	Released Cultivar	18.83	4339.56
R097	N24480.000	Released Cultivar	21.75	9923.73
R100	N25461	Released Cultivar	20.51	3082.75
R117	N10125.100	Released Cultivar	23.93	14937.66
R119	T614689-90	Released Cultivar	23.24	31717.69
R121	N21478.000	Released Cultivar	20.54	11036.54
R124	N23696.000	Released Cultivar	22.94	9992.75
R136	N22308.000	Released Cultivar	23.05	640.62
R143	T614625-26	Released Cultivar	23.09	9047.69
R148	N23783.000	Released Cultivar	20.34	19171.38
R151	N24452.000	Released Cultivar	21.33	5739.33
R156	N25111	Released Cultivar	19.73	17641.27
R158	N06034.000	Released Cultivar	17.57	7967.99
R159	N25442	Released Cultivar	22.64	25590.72
R163	T614613-14	Released Cultivar	22.71	14099.27
R167	N07917.000	Released Cultivar	19.28	17040.34
R175	T614660-61	Released Cultivar	21.48	10935.01

Individual code	NJAU code	Accession type	Oil content (%)	Relative expression of *SWEET39* in seeds at 30 DAF
R191	N25314	Released Cultivar	22.85	10297.45
R192	N25318	Released Cultivar	22.96	5846.40
R193	N25383	Released Cultivar	20.86	12133.15
R194	N24458.000	Released Cultivar	21.73	11993.79

Table S5 The candidate genes with potential alterations in amino acid sequences in predicted lipid metabolism related KEGG (Kyoto Encyclopedia of Genes and Genomes) pathways.

Pathways	Gene Names
fatty acid elongation	*Glyma.15G046300*, *Glyma.12G075100*, *Glyma.06G214800*
lipid metabolism	*Glyma.08G102100*, *Glyma.09G191700*, *Glyma.09G186900*, *Glyma.15G046300*, *Glyma.12G081900*, *Glyma.08G085800*, *Glyma.12G075100*, *Glyma.06G214800*, *Glyma.08G084300*, *Glyma.08G102900*
fatty acid biosynthesis	*Glyma.08G102100*, *Glyma.08G084300*
lipid biosynthesis proteins	*Glyma.08G102100*, *Glyma.08G085800*, *Glyma.08G084300*
alpha-linolenic acid metabolism	*Glyma.09G191700*, *Glyma.08G102900*
linoleic acid metabolism	*Glyma.08G102900*
fatty acid degradation	*Glyma.09G191700*
pyruvate metabolism	*Glyma.08G096300*, *Glyma.09G193800*, *Glyma.12G079700*
starch and sucrose metabolism	*Glyma.06G184200*, *Glyma.20G026300*, *Glyma.20G026700*
glycerolipid metabolism	*Glyma.08G085800*
